# The Significance of Normal Pretreatment Levels of CA125 (<35 U/mL) in Epithelial Ovarian Carcinoma

**DOI:** 10.5041/RMMJ.10180

**Published:** 2015-01-29

**Authors:** Joseph Menczer, Erez Ben-Shem, Abraham Golan, Tally Levy

**Affiliations:** Division of Gynecologic Oncology, Department of Obstetrics and Gynecology, E. Wolfson Medical Center, Holon, Tel Aviv University, Sackler Faculty of Medicine, Tel Aviv, Israel

**Keywords:** Epithelial ovarian cancer, normal CA125 levels, prognostic factors, survival

## Abstract

**Objective:**

To assess the association between normal CA125 levels at diagnosis of epithelial ovarian carcinoma (EOC) with prognostic factors and with outcome.

**Methods:**

The study group consisted of histologically confirmed EOC patients with normal pretreatment CA125 levels, and the controls consisted of EOC patients with elevated (≥35 U/mL) pretreatment CA125 levels, diagnosed and treated between 1995 and 2112. Study and control group patients fulfilled the following criteria: 1) their pretreatment CA125 levels were assessed; 2) they had full standard primary treatment, i.e. cytoreductive surgery and cisplatin-based chemotherapy; and 3) they were followed every 2–4 months during the first two years and every 4–6 months thereafter.

**Results:**

Of 114 EOC patients who fulfilled the inclusion criteria, 22 (19.3%) had normal pretreatment CA125 levels. The control group consisted of the remaining 92 patients with ≥35 U/mL serum CA125 levels pretreatment. The proportion of patients with early-stage and low-grade disease, with optimal cytoreduction, and with platin-sensitive tumors was significantly higher in the study group than in the control group. The progression-free survival (PFS) and overall survival (OS) were significantly higher in the study group than in the control group on univariate analysis but not on multivariate analysis.

**Conclusion:**

It seems that a normal CA125 level at diagnosis in EOC may also be of prognostic significance for the individual patient.

## INTRODUCTION

Based on the initial report by Bast et al.,^1^ the serum level of 35 U/mL is considered as the cutoff point for CA125. The serum level of CA125 is widely used for distinguishing malignant from benign pelvic masses, for monitoring the response of epithelial ovarian cancer (EOC) to treatment, for follow-up, and for detecting disease recurrence. However, this marker has not proved a useful EOC screening test because of its low sensitivity and specificity.^2^,^3^ Thus, serum levels of CA125 may be in the normal range in 50% of symptomatic stage I patients and in about 10%– 20% of advanced-stage patients.^3^–^5^ Elevated CA125 levels are most common in patients with serous ovarian carcinoma (SOC), while the other types of EOC often present with normal serum CA125 levels.^5^

Current standard treatment for ovarian cancer consists of cytoreductive surgery, followed by platinum-taxane based combination chemotherapy.^6^ Well-known prognostic factors of EOC include stage, grade, and optimal surgical cytoreduction.^7^ The recent meaning of “optimal” cytoreduction connotes actually complete tumor resection, i.e. no gross residual tumor.^8^,^9^ The presence of platin sensitivity is also a prognostic factor.^10^ To stratify patients for optimal therapy, additional prognostic and predictive factors are needed.

Investigations dealing specifically with the prognostic significance of normal pretreatment serum levels in EOC patients are scarce.^11^,^12^

The purpose of the present study was to assess the association between normal (<35 U/mL) pretreatment CA125 levels with prognostic factors and outcome in EOC patients.

## MATERIAL AND METHODS

The study group consisted of histologically confirmed EOC patients with normal pretreatment CA125 levels diagnosed and treated between 1995 and 2012. The controls consisted of EOC patients diagnosed during the same period with elevated (≥35 U/mL) pretreatment CA125 levels. The CA125 levels were tested in the same laboratory. Study and control group patients fulfilled the following inclusion criteria: 1) their pretreatment CA125 levels were assessed shortly before diagnosis and treatment; 2) they had full standard primary treatment; and 3) they were followed every 2–4 months during the first two years and every 4–6 months thereafter.

The records of the study and control group patients were retrospectively abstracted after institutional review board approval, and their clinicopathological data were recorded. All patients had primary cytoreductive surgery and surgical staging. Adjuvant chemotherapy consisted of intravenous paclitaxel (175 mg/m^2^) and carboplatin (AUC 6) for six 21-day cycles. Patients in whom recurrence occurred more than 6 months after completion of treatment were considered platin-sensitive.

Comparisons of the variables between patients with normal and elevated CA125 levels at diagnosis of EOC were made using the chi-square test for categorical variables and Fisher’s exact test, for small cells. Survival status was updated to September 2012. Overall survival (OS) and progression-free survival (PFS) were assessed using the Kaplan–Meier method, and comparison of survival between patients with normal and elevated CA125 was made by the log-rank test. Factors found to be significantly associated with survival on univariate analysis were evaluated using Cox proportional hazards regression testing.

## RESULTS

During the study period 114 EOC patients who fulfilled the inclusion criteria were diagnosed. Among these, 22 (19.3%) patients had pretreatment CA125 levels within normal limits. They comprised 13 SOC and nine non-SOC patients. The latter consisted of five endometrioid and two each mucinous and clear cell tumors. The control group consisted of the remaining 92 patients with elevated serum CA125 levels. They comprised 69 SOC and 23 non-SOC patients. The latter consisted of 15 endometrioid, six mucinous, and two clear cell tumors. Adjuvant chemotherapy was given to 17 (77.3%) study group patients and to 89 (96.7%) controls. There was no statistically significant difference in the mean age between the study group and the control group (62.8±9.0 and 59.6±10.1 years, respectively). The mean CA125 level in the study group was 18.8±10.3 U/mL and that of the control group 1,357±1,100 U/mL. Additional selected characteristics of the study and control group patients according to CA125 level are presented in Table 1. The proportion of patients with stage I, grade 1, optimal cytoreduction, and platin-sensitive tumors was significantly higher in the study group than in the control group.

**Table 1.** Selected Characteristics of the Patients According to Pretreatment CA125 Levels.

	Pretreatment Serum CA125 Level				

	<35 U/mL		≥35 U/mL		*P*
	
	No.	%	No.	%	
**Total**	22	100.0	92	100.0	
**Stage**					0.002
I	11	50.0	17	18.5	
II–IV	11	50.0	75	81.5	
**Grade**					0.01
1	7	31.8	10	10.9	
2, 3	15	68.2	82	89.1	
**Gross residual disease present**					0.001
Yes	3	13.6	48	52.2	
No	19	86.4	44	47.8	
**Platin sensitivity**					0.04
Yes	17	100.0	74	80.4	
No	0	0.0	18	19.6	

**Figure 1.**
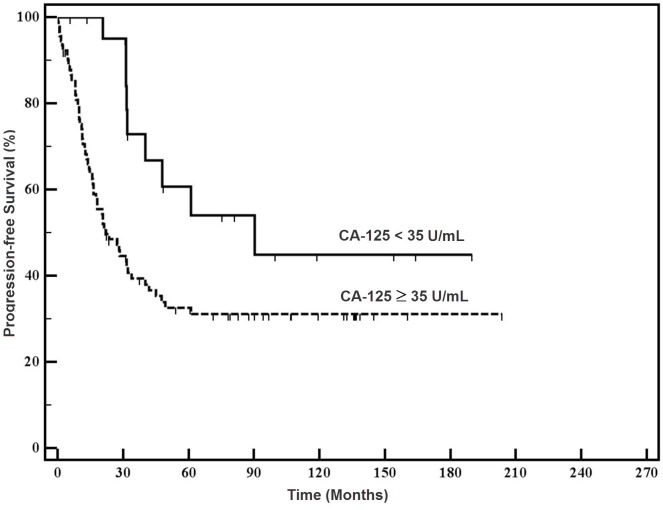
**Progression-Free Survival of the Study Patients and Controls.**

**Figure 2.**
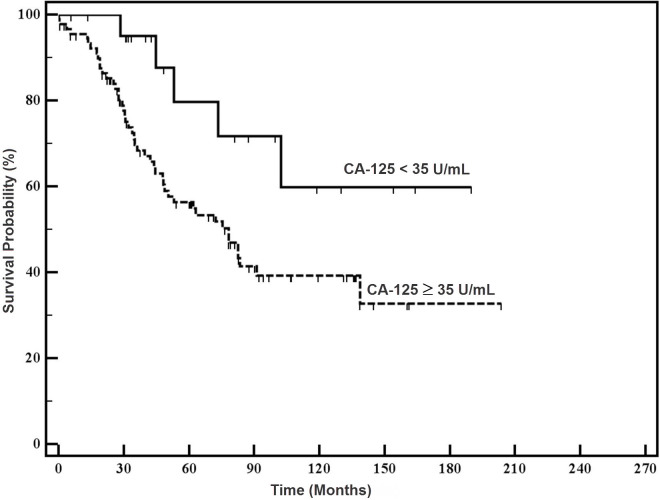
**Overall Survival of the Study Patients and Controls.**

The median PFS in the study group was significantly higher than in the control group (90.2 and 22.7 months, respectively; *P*=0.016, HR 2.3, 95% CI 1.33–3.97) (Figure 1). The median OS in the study group was significantly higher than in the control group (not reached versus 78.1 months, respectively; *P*=0.033, HR 2.6, 95% CI 1.37–5.08) (Figure 2). On multivariate analysis only stage and grade were independent prognostic factors.

## DISCUSSION

Our data indicate that normal pretreatment serum CA125 levels are associated with favorable prognostic factors and outcome in EOC patients.

Some issues concerning pretreatment CA125 are still controversial. In some studies it has been found that preoperative levels may reflect tumor burden,^4^,^13^ while others found no such correlation.^14^ In many studies it has also been shown that the preoperative level of CA125 can serve as a predictor of optimal tumor cytoreduction in advanced ovarian cancer.^15^–^19^ In these studies high preoperative CA125 serum levels were associated with decreased likelihood of achieving an optimal cytoreduction, although the threshold cutoff levels differed in them. On the other hand, in other studies preoperative serum CA125 level was not found to be a reliable predictor of optimal cytoreduction.^20^–^25^

Several studies included data regarding low preoperative CA125 levels (<100 U/mL) in the assessment of the ability to predict optimal cytoreduction and outcome. Vorgias et al.^26^ found that all of 19 patients with CA125 levels of <100 U/mL were optimally cytoreduced. Saygili et al.^15^ reported that at a serum level of <100 U/mL 80% of patients had optimal cytoreduction. According to Cooper et al.^21^ at the serum level of ≤61 U/mL the sensitivity and specificity for optimal cytoreduction were 15% and 98%, respectively, and the positive and negative predictive values were 93% and 37%, respectively. It was also reported that preoperative CA125 levels of <65 U/mL correlate with a better survival in EOC patients.^27^,^28^ In this context it is noteworthy that in patients with borderline ovarian tumors with CA125 levels lower than 50 U/mL the PFS and the OS were also found to be significantly better than in those with higher levels.^29^

We could locate only two studies that assessed specifically EOC patients with normal pretreatment CA125 levels. In a study that assessed only stage I patients, Paramasivam et al.^11^ found that the five-year overall survival rate was significantly higher for 176 patients with CA125 levels of <30 U/mL than in 342 patients with higher CA125 levels. Gundogdu et al.^12^ reported that significantly fewer stage III and IV patients with normal CA125 levels developed recurrence compared to those with higher levels.

We found that on univariate analysis normal pretreatment CA125 levels in EOC patients are significantly associated with lower stage and grade, with optimal cytoreduction, and with platin sensitivity. In view of the association between normal pretreatment serum CA125 levels with these favorable prognostic factors in our patients, the PFS and OS were also better than in those with elevated CA125 levels in univariate analysis. But, as could be expected, on multivariate analysis only stage and grade were independent prognostic factors. Nevertheless, it seems that a normal CA125 level at diagnosis of EOC is also of prognostic significance.

The main limitation of our study is the small number of EOC patients with normal CA125 levels pretreatment. Larger studies seem to be indicated.

## Abbreviations:

EOC: epithelial ovarian cancer;
OS: overall survival;
PFS: progression-free survival;
SOC: serous ovarian carcinoma.
